# The Effect of Phase Change Material on Recovery of Neuromuscular Function Following Competitive Soccer Match-Play

**DOI:** 10.3389/fphys.2019.00647

**Published:** 2019-06-06

**Authors:** Callum G. Brownstein, Paul Ansdell, Jakob Škarabot, Malachy P. McHugh, Glyn Howatson, Stuart Goodall, Kevin Thomas

**Affiliations:** ^1^Faculty of Health and Life Sciences, Northumbria University, Newcastle upon Tyne, United Kingdom; ^2^Univ Lyon, UJM-Saint-Etienne, Laboratoire Interuniversitaire de Biologie de la Motricité, Saint-Etienne, France; ^3^Nicholas Institute of Sports Medicine and Athletic Trauma, Lenox Hill Hospital, New York, NY, United States; ^4^Water Research Group, School of Environmental Sciences and Development, Northwest University, Potchefstroom, South Africa

**Keywords:** central nervous system, cryotherapy, fatigue, peripheral, recovery

## Abstract

**Aim:** Cryotherapy is commonly implemented following soccer match-play in an attempt to accelerate the natural time-course of recovery, but the effect of this intervention on neuromuscular function is unknown. The aim of the present study was to examine the effect of donning lower-body garments fitted with cooled phase change material (PCM) on recovery of neuromuscular function following competitive soccer match-play.

**Methods:** Using a randomized, crossover design, 11 male semi-professional soccer players wore PCM cooled to 15°C (PCM_cold_) or left at ambient temperature (PCM_amb_; sham control) for 3 h following soccer match-play. Pre-, and 24, 48, and 72 h post-match, participants completed a battery of neuromuscular, physical, and perceptual tests. Maximal voluntary contraction force (MVC) and twitch responses to electrical (femoral nerve) and magnetic (motor cortex) stimulation (TMS) during isometric knee-extension and at rest were measured to assess central nervous system (CNS) (voluntary activation, VA) and muscle contractile (quadriceps potentiated twitch force, Q_tw,pot_) function. Fatigue and perceptions of muscle soreness were assessed via visual analog scales, and physical function was assessed through measures of jump [countermovement jump (CMJ) height and reactive strength index (RSI)] performance. A belief questionnaire was completed pre- and post-intervention to determine the perceived effectiveness of each garment.

**Results:** Competitive soccer match-play elicited persistent decrements in MVC, VA measured with femoral nerve stimulation, Q_tw,pot_, as well as reactive strength, fatigue and muscle soreness (*P* < 0.05). Both MVC and VA were higher at 48 h post-match after wearing PCM_cold_ compared with PCM_amb_ (*P* < 0.05). However, there was no effect of PCM on the magnitude or time-course of recovery for any other neuromuscular, physical function, or perceptual indices studied (*P* > 0.05). The belief questionnaire revealed that players perceived that both PCM_cold_ and PCM_amb_ were moderately effective in improving recovery, with no difference between the two interventions (*P* = 0.56).

**Conclusion:** Although wearing cooled PCM garments improved MVC and VA 48 h following match-play, the lack of effect on measures of physical function or perceptual responses to match-play suggest that PCM offers a limited benefit to the recovery process. The lack of effect could have been due to the relatively small magnitude of change in most of the outcome measures studied.

## Introduction

Association football (soccer) is an intermittent-sprint sport which imposes high physiological, neuromuscular and cognitive demands (Mohr et al., 2005). During a typical match, players cover 10–13 km, with 2–3 km covered at high intensities, and a diverse range of high-intensity movements performed, such as accelerating, decelerating, changing direction, impacts and tackles (Mohr et al., 2003). An inexorable consequence of these demands is fatigue, defined as a sensation of tiredness and weakness underpinned and/or modulated by a multitude of physiological and psychological processes (Thomas et al., 2018). The fatigue which occurs as a result of soccer match-play persists post-exercise, and can take days to resolve (Rampinini et al., 2011). Nevertheless, in most top professional leagues, it is normal procedure for teams to compete in three successive games during a 7 days period at several stages throughout a season, often with as little as 48–72 h recovery between games. Due to the demanding nature of soccer match-play and the congested fixture schedules in the modern-day game, understanding the etiology of fatigue, the time-course of recovery, and strategies to alleviate fatigue and expedite recovery are pertinent issues (Nedelec et al., 2012, 2013).

When implementing recovery strategies aimed at alleviating fatigue and accelerating recovery, it is imperative to understand the stressors causing reductions in performance and delayed recovery before applying the intervention (Howatson et al., 2016). While the fatigue which persists in the days following soccer match-play is multifactorial and complex, impairment in maximal voluntary contraction (MVC) strength, which can take up to 72 h to resolve (Brownstein et al., 2017), is likely an important contributor to post-match fatigue. In turn, impairments in MVC strength are underpinned by a multitude of processes, and are often attributed to impairments in neuromuscular function, measured as deficits in contractile function and/or the capacity of the central nervous system (CNS) to activate muscle (Gandevia, 2001). Using neurostimulation techniques, a recent study from our laboratory examined the effect of soccer match-play on neuromuscular function in the days post-match, and demonstrated substantial impairments in contractile and CNS function which required up to 48 h to recover (Brownstein et al., 2017). In turn, it was further hypothesized that the protracted impairments in contractile and CNS function were likely a consequence of the repeated eccentric contractions associated with match-play and the subsequent muscle damage and inflammatory response which ensues (Ascensao et al., 2008; Brownstein et al., 2017). A number of factors would support this suggestion. Firstly, it is known that soccer match-play induces considerable muscle damage and a prolonged inflammatory response which can persist for several days post-exercise (Ispirlidis et al., 2008; Fatouros et al., 2010). Secondly, while impairments in contractile and CNS function can also occur due to metabolic influences (Allen et al., 2008), many of the metabolic mechanisms thought to interfere with neuromuscular function dissipate rapidly following exercise cessation. For example, following exercise that imposes large metabolic but little mechanical demand, recovery is substantially faster than exercise that is mechanically demanding (Skurvydas et al., 2016). In addition, the mechanical stress imposed on muscle fibers during eccentric based exercise has been shown to elicit prolonged impairments in the excitation-contraction coupling process (Souron et al., 2018), as well as residual deficits in voluntary activation which can take days to resolve (Goodall et al., 2017). As such, it is a plausible assumption that the impaired neuromuscular function which persists for several days following soccer match-play is primarily a consequence of muscle damage and the associated inflammatory response, and strategies to alleviate the negative effects of muscle damage and inflammation could thus be suitable to accelerate recovery following competitive soccer match-play.

The precise mechanisms of exercise-induced muscle damage (EIMD) are complex and remain to be fully elucidated. However, muscle damage has previously been simplified into two general areas; the initial event that occurs during the exercise bout (termed “primary damage”), and the secondary events that propagate damage through factors associated with inflammation (termed “secondary damage”) (Howatson and van Someren, 2008; Owens et al., 2018). While the inflammatory response that ensues following EIMD is thought to be crucial in orchestrating muscle repair and recovery (Butterfield et al., 2006), the secondary damage associated with inflammation is suggested to further exacerbate impairments in muscle function (Pizza et al., 2005). As such, a common target of interventions is to alleviate the negative effects associated with the inflammatory response in an attempt to expedite the recovery process (Howatson et al., 2010; Rowsell et al., 2011).

A common post-exercise recovery strategy is cryotherapy, which is regularly implemented following soccer match-play, and is supposed to attenuate post-exercise reductions in functional capacity and athletic performance (Nedelec et al., 2013). While the precise underlying mechanisms remain to be elucidated, cryotherapy is purported to reduce muscle temperature and attenuate inflammation and oxidative stress (White and Wells, 2013). A recently implemented form of cryotherapy that has produced encouraging results as a recovery aid is phase change material; PCM (Clifford et al., 2018; Kwiecien et al., 2018; McHugh et al., 2018). Phase change material is a substance with a high heat fusion, which melts and solidifies at certain temperatures. When frozen PCM is convectively heated, for example, through exposure to the human body, it will continuously absorb heat until all material has changed from solid to liquid. As such, PCM can maintain low temperatures within the tissues of the target limb for sustained periods. The application of PCM has many logistical and practical benefits due to being easily transportable, the lower level of thermal discomfort compared with cryotherapy, and capacity to maintain low temperatures for a prolonged period of time (Kwiecien et al., 2018). A recent study applied cold PCM to the quadriceps for 3 h following competitive soccer match-play and found reduced muscle soreness and accelerated recovery of MVC (Clifford et al., 2018), findings which have since been corroborated (McHugh et al., 2018).

Despite the promising results of recent studies (Clifford et al., 2018; Kwiecien et al., 2018; McHugh et al., 2018), more evidence is required to substantiate the efficacy of PCM as a recovery intervention and to gain mechanistic insight into the potential benefits of PCM on recovery. Accordingly, the aim of the present study was to examine the effect of wearing cold PCM garments on recovery of neuromuscular function, as well as physical and perceptual measures following soccer match-play. It was hypothesized that wearing cold PCM garments would expedite recovery of impaired neuromuscular function and attenuate muscle soreness, possibly by reducing the negative effects associated with the acute inflammatory response on contractile and CNS function.

## Materials and Methods

### Participants

After receiving ethical approval from the Northumbria University Faculty of Health and Life Sciences Ethics committee in accordance with the ethical standards established in the Declaration of Helsinki, fifteen male semi-professional soccer players from Level eight of the English Football League, gave written informed consent to participate in the study. Throughout the data collection period, four players sustained injuries which prevented them from completing the study, leaving eleven participants in total (three defenders, five midfielders, three attackers; 22 ± 1 years; stature 1.80 ± 0.10 m; mass 78 ± 8 kg). Players trained three to four times a week, in addition to at least one competitive match. The participants competitive season ran from August to May, with testing taking place in the mid-season phase of the players training year. Participants were required to refrain from physical activity and alcohol consumption for the duration of the study and in the 48 h prior to data collection and abstain from caffeine consumption for the 12 h prior to each experimental visit.

### Design

The study employed a randomized cross-over design to assess the effectiveness of PCM on recovery in the days following competitive soccer match-play. Participants visited the laboratory prior to commencement of the data collection period for habituation to the measurement tools employed in the study. For the experimental trials, participants were required to visit the laboratory prior to and 24, 48, and 72 h following two competitive soccer matches. The pre-match visit took place 24 h before the fixtures. On one occasion, players wore shorts fitted with PCM (Glacier Tek; USDA BioPreferred PureTemp, Plymouth, MN) that was either cooled (PCM_cold_) or left ambient (PCM_amb_), which served as a sham control. The order of the conditions was randomized using an online randomizer^1^. Phase change material was applied to the quadriceps and hamstring muscle groups, and was worn for 3 h post-match. To ensure compliance with the intervention, away fixtures in which the team were required to travel back for ≥3 h were selected. The two fixtures were separated by 4–8 weeks. During each experimental visit, participants completed assessments of neuromuscular, physical, and perceptual function to ascertain the effect of PCM on recovery.

### Procedures

#### Practice Trial

Prior to the experimental trials, participants attended the laboratory for habituation with the study procedures. This involved an explanation of the methods employed in the study, before participants performed a practice trial consisting of the neuromuscular, physical and perceptual measures employed in the study (described below).

### Experimental Trials

#### Competitive Soccer Match

Participants visited the laboratory 24 h prior to each match for pre-match measurements (described in detail below). On the subsequent day, players completed a 90 min soccer match within their competitive league consisting of two 45 min halves interspersed by a 15 min recovery interval. In total, the study took place across six matches, with five participants investigated following games one and two, three participants investigated following games three and four, and three participants investigated following games five and six. All fixtures took place on a grass pitch at either 13:00 (games one, two, and six) or 14:00 (games three, four, and five). Players were required to play a minimum of 70 min per match in order to be included in the experiment. The activity profiles and heart rates of the players were measured throughout the games using GPS with built in heart rate monitors (Polar Team Pro, Polar Electro Oy, Finland), and compared between games in order to ensure the physical and physiological demands of the matches in each condition were similar.

#### Phase Change Material

Prior to the post-match application of PCM_cold_, the temperature of the blocks was cooled and maintained in a freezer at 15°C, while PCM_amb_ were stored >22°C. When traveling to the fixtures, PCM_cold_ were stored in an insulated storage container. The PCM blocks worn over the quadriceps were 32 cm in length and 13 cm in width, while the blocks worn over the hamstrings were 16 cm in length and 13 cm in width. Two blocks were worn on the quadriceps and hamstring muscles inside compression shorts, with blocks placed over the medial and lateral parts of both muscle groups. The PCMs were applied within 30 min post-exercise, and were worn while traveling back from the matches on the team bus.

#### Outcome Measures

A range of neuromuscular, physical and perceptual measures were assessed 24 h pre-match, and 24, 48, and 72 h post-match. Details of these measures are provided below.

#### Perceptual Responses

Participants completed the “*Elite Performance Readiness Questionnaire”* (Dean et al., 1990) at each time point, a measure of performance readiness consisting of 10 subjective measures of fatigue, soreness, motivation to train, anger, confusion, depression, tension, alertness, confidence, and sleep. Participants drew a vertical line on a 100 mm horizontal line in response to questions used for each measure, such as “how fatigued do you feel?” “how sore do your muscles feel?” and “how motivated to train do you feel?” Each scale was anchored with verbal descriptors “not at all” to “extremely.” Perceptual measures were assessed at each time-point prior to commencing the warm-up. In addition, similar to a previous study (Clifford et al., 2018) participants completed a questionnaire in which they rated how effective they felt the cold and ambient PCM were going to be for recovery prior to the intervention (pre-match), and how effective they felt they were in improving recovery at the end of the intervention (72 h post-match). The belief questionnaire consisted of a Likert scale from 1 “not effective at all” to 5 “extremely effective.”

#### Assessment of Neuromuscular Function

Measures of neuromuscular function were assessed at each time-point with electrical stimulation of the femoral nerve and TMS of the contralateral motor cortex at rest and during voluntary contractions of the right knee-extensors. The neuromuscular assessment began with two practice MVCs to ensure potentiation of subsequent evoked measures, followed by three ∼3 s MVCs, all separated by 30 s. During these 3 MVCs, paired motor nerve stimulation (100 Hz) was delivered when peak force plateaued, and ∼2 s after the MVC to measure voluntary activation (VA), with a single pulse electrical stimuli delivered 5 s post-MVC to assess potentiated quadriceps twitch force (Q_tw,pot_) of the knee-extensors. The average of the 3 MVCs was included in the analysis. Single-pulse TMS was subsequently delivered during two sets of five 3–5 s contractions at 100, 87.5, 75, 62.5, and 50% MVC, with 5 s rest between contractions and 10 s rest between sets, to determine VA_TMS._

##### Force and electromyographical recordings

The evoked quadriceps force and electromyographic (EMG) responses of the *rectus femoris* (RF) to TMS of the primary motor cortex, and electrical stimulation of the femoral nerve, were used to assess neuromuscular function. A calibrated load cell (MuscleLab force sensor 300, Ergotest technology, Norway) recorded muscle force (N) during an isometric voluntary contraction of the knee extensors. During contractions, participants sat with hips and knees at 90° flexion, with a load cell fixed to a custom-built chair and attached to the participants right leg, superior to the ankle malleoli, with a noncompliant cuff. Electromyographic activity from the RF and *biceps femoris* (BF) was recorded from surface electrodes (Ag/AgCl; Kendall H87PG/F, Covidien, Mansfield, MA, United States) placed 2 cm apart over the belly of each muscle, with a reference electrode placed on the patella. The placement of the EMG electrodes was based on SENIAM guidelines (Hermens et al., 2000). Electrode placement was marked with indelible ink to ensure consistent placement throughout the study, with the areas cleaned and shaved prior to electrode placement. The electrodes recorded electrical activity in the RF and BF, with the signal processed to permit analysis of the root-mean-square (RMS) amplitude for sub-maximal and MVCs, the maximal compound muscle action potential (M_max_) from the electrical stimulation of the femoral nerve, and the motor evoked potential (MEP) elicited by TMS. Signals were amplified: gain × 1,000 for EMG and × 300 for force (CED 1902; Cambridge Electronic Design, Cambridge, United Kingdom), band-pass filtered (EMG only: 20–200 Hz), digitized (4 kHz; CED 1401, Cambridge Electronic Design) and analyzed offline. Further details on these methods are provided below.

##### Motor nerve stimulation

Motor nerve stimulation was used for the measurement of contractile function, muscle membrane excitability and VA. Single and paired electrical stimuli (100 Hz) were administered using square wave pulses (200 μs) via a constant-current stimulator (DS7AH, Digitimer Ltd., Hertfordshire, United Kingdom) using self-adhesive surface electrodes (CF3200, Nidd Valley Medical Ltd., North Yorkshire, United Kingdom). Electrical stimuli were first administered to the motor nerve at rest in 20 mA step-wise increments from 20 mA until the maximum quadriceps twitch amplitude (Q_tw_, N) and M_max_ (mV) were elicited. To ensure a consistent, supramaximal stimulus and account for any activity-induced changes in axonal excitability, the resulting stimulation intensity was increased by 30% (198 ± 38 mA). The peak-to-peak amplitude and area of the electrically evoked maximal compound action potential (M_max_) was used as a measure of membrane excitability. In addition, the following mechanical measures of muscle contractility were derived from the single pulse potentiated twitch response: contraction time (CT, time to peak twitch tension), maximum rate of force development (MRFD, maximal linear incline of the force response calculated at 100 ms epochs), maximal rate of relaxation (MRR, maximal linear decline of the force response calculated at 100 ms epochs), and one half relaxation time.

##### Voluntary activation with TMS

Single-pulse TMS was delivered over the motor cortex via a concave double cone coil using a Magstim 200^2^ stimulator (The Magstim Company Ltd., Whitland, United Kingdom). The junction of the double cone coil was aligned tangentially to the sagittal plane, with its center 1–2 cm to the left of the vertex and was oriented to induce current in the posterior-to-anterior direction. The optimal coil placement was determined at the start of each trial as the position that elicited the largest MEP in the RF, with a concurrent small MEP in the BF during a light voluntary contraction (10% MVC). The optimal position was marked with indelible ink to ensure consistent placement throughout the study. To determine VA with TMS (VA_TMS_), single pulse TMS was delivered during brief (3–5 s) contractions at 100, 87.5, 75, 62.5, and 50% MVC, separated by 5 s of rest (Dekerle et al., 2018). This procedure was repeated two times, with 15 s between each set. The stimulation intensity was set at the stimulator output that elicited the maximum superimposed twitch force during a 50% MVC (Thomas et al., 2017), and did not differ between conditions (PCMcold 66 ± 10% vs. PCMamb 68 ± 8%, respectively, *P* = 0.57), or across the 4 time-points (*P* = 0.49). The stimulator output activated a large proportion of the KE motoneuron pool at baseline, with no difference between PCM_cold_ (67 ± 24% M_max_ amplitude) or PCM_amb_ (61 ± 13%, *P* = 0.38). Small co-activation of the antagonist muscle (BF) was observed in response to TMS and did not differ between PCM_cold_ (0.85 ± 0.37 mV) or PCM_amb_ (0.86 ± 0.36 mV, *P* = 0.96) or across the 4 time-points (*P* = 0.51).

#### Assessment of Physical Function

Participants completed a battery of assessments to measure physical function in variables relevant to optimal soccer performance. All measures of physical function were performed following the neuromuscular assessment and the completion of a standardized warm-up. An optical timing system (Optojump Next, Microgate, Milan, Italy) was used to measure jump height (cm) during a countermovement jump (CMJ), and reactive strength index (RSI) during a drop jump (DJ). For CMJ, participants started from an erect position with hands akimbo. On verbal command, participants made a downward countermovement before jumping vertically for maximum height. For reactive strength index (DJ-RSI), participants were instructed to step off a 30 cm box, before jumping vertically for maximum height as soon as possible after landing, maintaining hands akimbo throughout. To ensure the DJ-RSI was assessing fast stretch-shortening cycle function, a maximum ground contact time of 200 ms was allowed during each jump, with participants given visual feedback on each ground contact time and jump height after each jump (Thomas et al., 2017). Reactive strength index (cm ⋅ s^-1^) was calculated as the ratio between jump height (cm) and ground contract time (s). All participants were given three attempts at each jump with 60 s between each repetition. For CMJ and DJ-RSI, the best of the three attempts was included in the analysis.

#### Match-Play Physical Performance and Intensity

During the games, GPS with built in HR monitors (Polar Team Pro, Polar Electrophase Oy, Finland) were used to assess total distance (TD), high-intensity running (HIR, distance covered at running velocities higher than 15 km ⋅ h^-1^), total accelerations (>1 m ⋅ s^-2^), total decelerations (> -1 m ⋅ s^-2^), and mean and peak HR (Akenhead et al., 2013). These variables were compared between games to ensure the physical and physiological demands of the matches in each condition were similar.

### Data Analysis

Voluntary activation was assessed through the interpolated twitch technique and was quantified by comparing the amplitude of the superimposed twitch force (SIT) with the potentiated twitch force (100 Hz) delivered 2 s following the MVC at rest using the following equation: Motor nerve VA (%) = [1 - (SIT/Q_tw,pot_) × 100]. VA_TMS_ was assessed during two sets of contractions at 100, 87.5, 75, 62.5, and 50% MVC according to Dekerle et al. (2018), and the regression between SIT amplitude and contraction intensity was extrapolated to the y intercept to obtain an estimated resting twitch (ERT, Todd et al., 2003). The regression analysis confirmed a linear relationship at each time-point (*r*^2^ range = 0.89 ± 0.04–0.93 ± 0.06). The estimated resting twitch (ERT) was calculated as the *y*-intercept of the linear regression between the mean amplitude of the SIT force evoked by TMS at each contraction intensity. Subsequently, VA_TMS_ was quantified using the equation [1 - (SIT/ERT) × 100]. The peak-to-peak amplitude of evoked MEP and M_max_ were measured offline.

### Reproducibility Coefficients

Typical error as a coefficient of variation (CV, %) and intraclass correlation coefficients (ICC_3,1_) between the two baseline visits were calculated to quantify the reproducibility of neuromuscular and physical function measures. Reproducibility coefficients were as follows: MVC (ICC = 0.97, CV = 1.7%), VA with motor nerve stimulation (ICC = 0.85, CV = 2.8%), M_max_ (ICC = 0.91, CV = 4.3%), Q_tw,pot_ (ICC = 0.84, CV = 4.3%), VA_TMS_ (ICC = 0.81, CV = 4.1%),MRFD (ICC = 0.89, CV = 0.9%), MRR (ICC = 0.80, CV = 2.6%), CT (ICC = 0.60, CV = 8.1%), and RT_0.5_ (ICC = 0.71, CV = 11.1%), CMJ (ICC = 0.97, CV = 6.6%), DJ-RSI (ICC = 0.87, CV = 7.7%).

### Statistical Analysis

Data are presented as mean ± SD. A two-way repeated measures ANOVA with 2 treatment levels (PCM_cold_ vs. PCM_amb_) with 4 time points (Pre-, 24, 48, and 72 h post-match) was performed. Normality of the data was assessed using the Shapiro–Wilks test. Assumptions of sphericity were explored and controlled for all variables using the Greenhouse-Geisser adjustment, where necessary. In the event of a significant interaction effect (treatment × time), Bonferonni *post hoc* analysis was performed to locate where the differences lie. Paired sample *t*-tests were used to assess differences in match-running and heart rate variables between the two conditions. The belief questionnaire was analyzed using the Wilcoxon signed-rank test. To estimate the magnitude of the treatment effects, Cohen’s *d* effect sizes (ES) were calculated with the magnitude of effects considered either small (0.20–0.49), medium (0.50–0.79), or large (>0.80). All data were analyzed using Statistical Package for Social Sciences (SPSS version 22.0). Statistical significance was accepted at *P* < 0.05.

## Results

### Match Performance and Intensity

**FIGURE 1:**
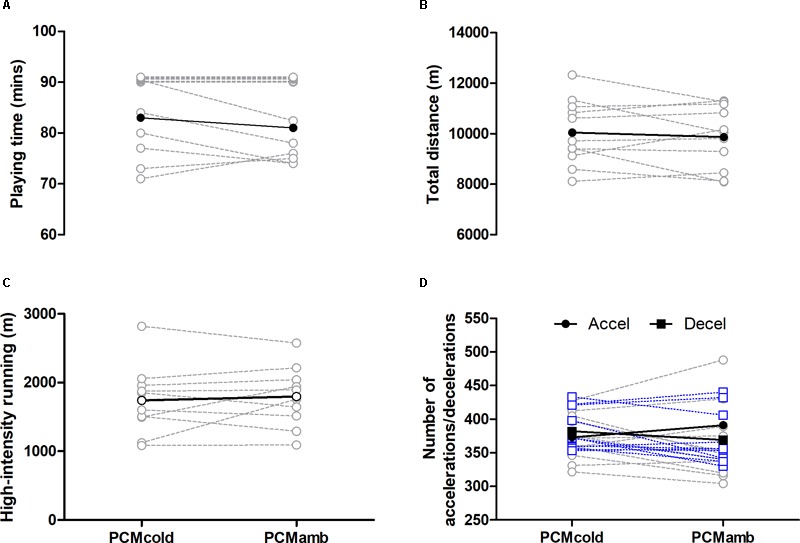
Playing time **(A)**, total running distance **(B)**, high-intensity running **(C)** and number of accelerations (dashed gray line) and decelerations (dashed blue line; **D**) measured during soccer match-play for two conditions (PCM_cold_ vs. PCM_amb_; *n* = 11). Values corresponding to 90 min in **(A)** are offset to prevent overlapping for clarity.

Match activity variables are displayed in Figure 1. No differences in playing time, match activity, or heart rate variables (mean HR 167 ± 9 and 165 ± 5, peak HR 192 ± 7 and 195 ± 9 for PCM_cold_ and PCM_amb_, respectively) were found between the two conditions (*P* ≥ 0.10). Players were required to play at least 70 min in order to be included in the intervention; no players were excluded on this criterion. In terms of treatment order, six players wore PCM_amb_ first and five players wore PCM_cold_.

### Perceptual Responses

Perceptual responses from the Elite Performance Readiness Questionnaire can be viewed in Table 1. Soccer match-play elicited fatigue [*F*_(3, 30)_ = 18.62, *P* < 0.001] and soreness [*F*_(3,30)_ = 17.99, *P* < 0.001] which persisted up to 72 h relative to baseline (all *P* ≤ 0.03). No effects of PCM were observed for any of the perceptual responses [*F*_(3, 30)_ = ≤ 0.65, *P* ≥ 0.59]. Analysis of the belief questionnaire revealed no differences in the perceived effectiveness of the two treatments either pre- or post-intervention (*P* = 0.56; Table 2).

**Table 1:** Perceptual responses measured through a visual analog scale (mm) at pre-, and 24, 48, and 72 h post-match (*n* = 11) for two conditions (PCM_cold_ vs. PCM_amb_).

	PCM_cold_				PCM_amb_			
	Pre-	24 h	48 h	72 h	Pre-	24 h	48 h	72 h
Fatigue	15.2 ± 11.8	55.5 ± 17.7^∗∗^	37.3 ± 21.7^∗^	23.7 ± 9.2	20.9 ± 18.0	51.7 ± 21.0^∗∗^	41.0 ± 13.6^∗^	24.0 ± 14.6
Soreness	18.6 ± 13.5	53.9 ± 17.7^∗∗^	40.2 ± 16.1^∗∗^	20.8 ± 18.3	23.5 ± 20.7	52.1 ± 19.6^∗∗^	51.8 ± 18.2^∗∗^	28.4 ± 19.1
Motivated to train	74.4 ± 20.2	51.6 ± 21.4	66.8 ± 14.4	67.6 ± 18.6	71.8 ± 23.6	45.2 ± 18.6	57.2 ± 24.5	64.8 ± 25.3
Anger	11.8 ± 9.4	12.9 ± 10.9	7.5 ± 4.5	7.7 ± 6.9	10.5 ± 9.7	14.6 ± 18.6	8.5 ± 7.1	7.1 ± 6.4
Confusion	18.6 ± 13.5	53.9 ± 17.7	40.2 ± 16.1	20.8 ± 18.3	23.5 ± 20.7	52.1 ± 19.6	51.8 ± 18.2	28.4 ± 19.1
Depression	8.5 ± 6.6	16.0 ± 17.2	8.1 ± 5.7	7.2 ± 4.8	7.7 ± 7.2	8.5 ± 6.6	8.9 ± 8.1	8.9 ± 6.2
Tension	20.5 ± 15.9	33.5 ± 25.1	17.6 ± 14.0	14.5 ± 8.6	18.9 ± 16.2	30.8 ± 22.9	25.0 ± 15.3	18.8 ± 15.6
Alertness	68.4 ± 16.7	46.5 ± 23.2	60.5 ± 17.0	63.9 ± 24.4	66.5 ± 22.4	54.5 ± 20.1	65.4 ± 18.8	65.6 ± 14.9
Confidence	65.6 ± 21.6	71.5 ± 12.3	66.8 ± 22.1	74.5 ± 10.6	71.9 ± 15.3	71.1 ± 12.0	70.7 ± 16.0	75.6 ± 12.5
Sleep	67.1 ± 18.1	63.5 ± 27.5	65.9 ± 18.1	64.2 ± 25.1	72.7 ± 24.4	56.5 ± 27.4	66.5 ± 15.2	63.5 ± 25.4

*Values are mean ± SD. Significant differences in comparison with baseline indicated by ^∗^p < 0.05, ^∗∗^p < 0.01.*

**Table 2:** Percieved effectiveness of the PCM garments for recovery before and after the intervention measured using a 1–5 Likert scale.

	PCM_cold_	PCM_amb_
Pre-match	3.6 ± 0.5	3.0 ± 0.6
72 h post-match	3.3 ± 0.9	3.0 ± 1.0

### Neuromuscular Function

**FIGURE 2:**
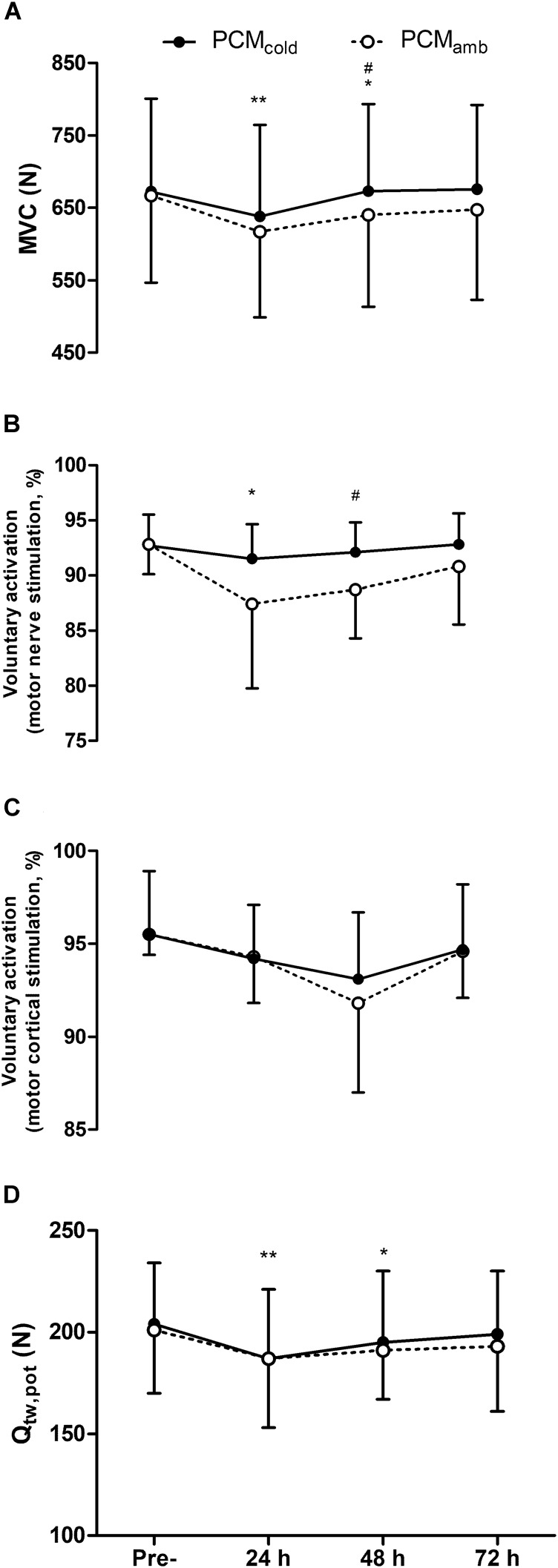
Maximal voluntary contraction force (MVC, **A**), voluntary activation measured with femoral nerve stimulation **(B)**, voluntary activation measured using motor cortical stimulation **(C)**, and quadriceps potentiated twitch force (Q_tw,pot_, **D**) measured at pre-, 24, 48, 72 h post-competitive soccer match-play for two conditions (PCM_cold_ vs. PCM_amb_; *n* = 11). Values are mean ± SD. Significant between-treatment differences indicated by ^#^*p* < 0.05. Significant differences in comparison with baseline indicated by ^∗^*p* < 0.05 and ^∗∗^*p* < 0.01.

**FIGURE 3:**
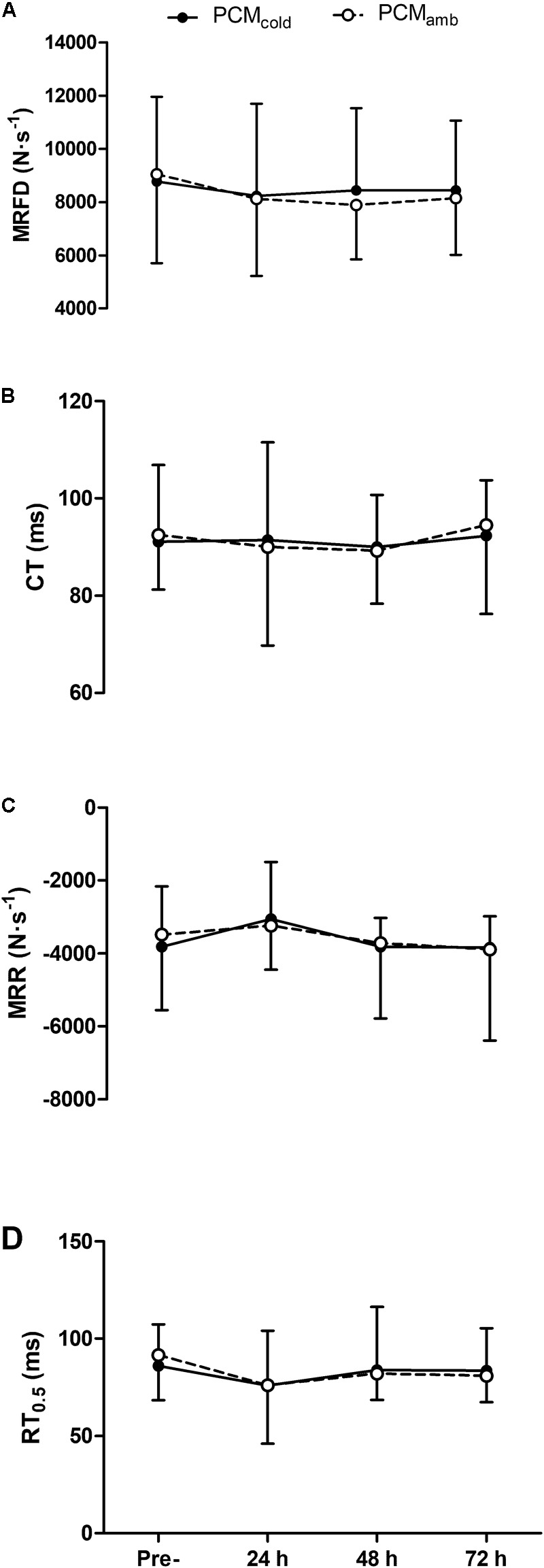
Maximum rate of force development (MRFD, **A**), contraction time (CT, **B**), maximum rate of relaxation (MRR, **C**) and half-relaxation time (RT_0.5_, **D**) measured from the electrically evoked quadriceps potentiated twitch (Q_tw,pot_) assessed at pre-, 24, 48, 72 h post-competitive soccer match-play for two conditions (PCM_cold_ vs. PCM_amb_; *n* = 11). Values are mean ± SD.

Neuromuscular function variables are depicted in Figure 2. Soccer match-play elicited declines in MVC force [*F*_(3, 30)_ = 6.26, *P* < 0.01], VA measured with motor nerve stimulation [*F*_(3,30)_ = 5.05, *P* < 0.01], and Q_tw,pot_ [*F*_(3, 30)_ = 3.09; *P* = 0.03], with impairments in MVC and Q_tw,pot_ persisting for up to 72 h post-match (all *P* ≤ 0.04), and reductions in VA persisting for up to 48 h post-match (*P* = 0.03). VA_TMS_ did not change at any time-point [*F*_(3, 30)_ = 2.662, *P* = 0.129]. Similarly, no time effect was found for M_max_ [range 4.4–4.8 mV, *F*_(3, 30)_ = 0.808, *P* = 0.524], or any measure of muscle contractility [*F*_(3, 30)_ ≤ 0.768, *P* ≥ 0.547; Figure 3]. No treatment × time interactions were observed for any of the neuromuscular variables (*F* ≤ 2.73, *P* ≥ 0.18). However, a main effect of treatment was found for both MVC [*F*_(1, 10)_ = 6.254, *P* = 0.03] and VA with motor nerve stimulation [*F*_(1, 10)_ = 5.47, *P* = 0.04]. *Post hoc* analyses revealed that for both MVC and VA, there was no difference between PCM_cold_ and PCM_amb_ at 24 h (MVC: *P* = 0.28; motor nerve VA: *P* = 0.61), while at 48 h, MVC (*P* = 0.03; *d* = 0.26) and VA (*P* = 0.01; *d* = 0.66) were higher in the PCM_cold_ compared with the PCM_amb_ condition.

### Physical Function

**FIGURE 4:**
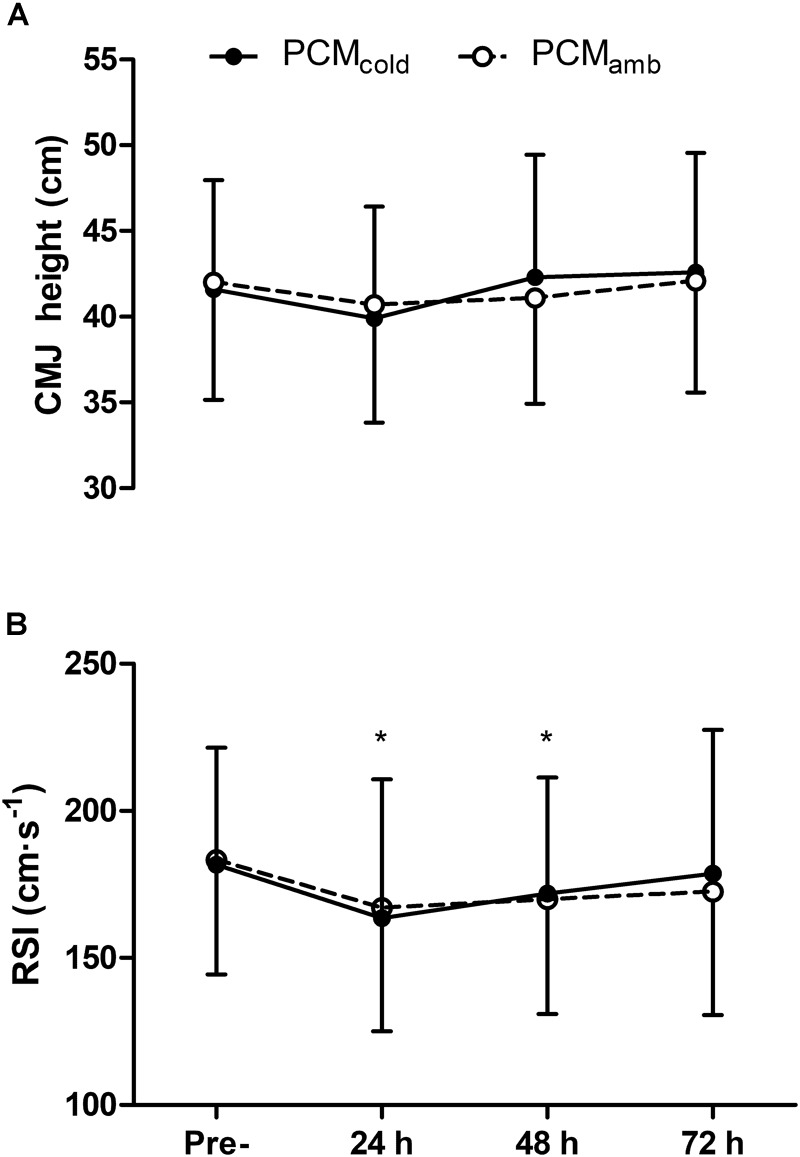
Countermovement jump height (CMJ, **A**) and reactive strength index (RSI, **B**) measured at pre-, 24, 48, 72 h post-competitive soccer match-play for two conditions (PCM_cold_ vs. PCM_amb_; *n* = 11). Values are mean ± SD. Significant differences in comparison with baseline indicated by ^∗^*p* < 0.05.

Physical function variables are displayed in Figure 4. Although a main effect for time on CMJ height was observed [*F*_(3, 30)_ = 5.01, *P* = 0.03], *post hoc* analysis revealed no significant differences relative to baseline (Figure 4A). Soccer match-play results in reductions in RSI [*F*_(3, 30)_ = 7.45, *P* = 0.02] which persisted for up to 48 h (*P* = 0.02; Figure 4B). There was no effect of PCM on any of the physical function variables [treatment × time *F*_(3,30)_ *≥* 1.05, *P* ≥ 0.20].

## Discussion

The aim of the present study was to examine the effect of wearing cold PCM garments on recovery of neuromuscular function, physical function and perceptual measures following soccer match-play. It was hypothesized that wearing cold PCM garments would expedite recovery of impaired neuromuscular function and attenuate muscle soreness, possibly by reducing the negative effects associated with the acute inflammatory response on contractile and CNS function. Although there was no difference in the change in any of the neuromuscular, physical function or perceptual indices over time, as indicated by the lack of a treatment × time interaction, MVC and VA were higher 48 h post-match after wearing PCM_cold_ compared with PCM_amb_, with the between-treatment differences at these time-points greater than the measurement error. Nevertheless, PCM_cold_ had no apparent effect on contractile function (measured through evoked responses to electrical stimulation at rest), physical function, and fatigue or soreness in the days following match-play. These findings suggest that while wearing cold PCM garments could attenuate the magnitude of impairments in MVC and the ability of the CNS to activate the knee extensors, the lack of effect on measures of physical performance or perceptual responses during the recovery period post-soccer match-play implies that PCM offers a limited benefit to the recovery process.

### Fatigue and Impairments in Neuromuscular Function Following Competitive Match-Play

The magnitude of impairments in the maximal force generating capacity of the muscle and the time-course of recovery in the present study was similar to that observed following competitive match-play in a study conducted by Rampinini et al. (2011), but less than was observed by Brownstein et al. (2017), in which MVC remained 11% below baseline at 24 h post. Specifically, MVC was reduced at 24 (PCM_cold_ 5.2%, PCM_amb_ 7.5%) and 48 h (PCM_amb_ 4.3%), before recovering by 72 h post-match. Similarly, Q_tw,pot_ was reduced at 24 (PCM_cold_ 8.0%, PCM_amb_ 6.7%), and 48 h (PCM_cold_ 4.2%, PCM_amb_ 3.4%), before recovering by 72 h post-match. Voluntary activation measured with motor nerve stimulation was reduced at 24 h (PCM_cold_ 1%, PCM_amb_ 6%) before recovering by 48 h post-match. In addition, physical function measured through the DJ-RSI was impaired for up to 72 h post-match, while analysis of perceptual responses indicate that fatigue and muscle soreness persisted for up to 72 h post-match. Furthermore, the reduction in MVC, one of the most widely used indicators of EIMD (Goodall et al., 2017), along with the increase in muscle soreness for up to 72 h post-match, indicates that the competitive soccer matches involved in the study elicited muscle damage. The occurrence of muscle damage was likely a consequence of the high volume of decelerations recorded throughout the matches along with the numerous other eccentric actions associated with soccer match-play. Given that recovery of contractile and CNS function has been shown to occur rapidly following exercise that is metabolically, but not mechanically demanding (Skurvydas et al., 2016), it is likely that the prolonged impairments in Q_tw,pot_ and VA in the present study were a consequence of the muscle damage incurred during match-play along with the inflammatory response which ensues thereafter.

### Effect of PCM_cold_ on Neuromuscular Function

The decline in the maximum force generating capacity of the quadriceps was attenuated following PCM_cold_, as indicated by the higher MVC at 48 h post compared with PCM_amb_. Furthermore, the between-treatment differences in the magnitude of reduction in MVC between baseline and 24 (2.3%) and 48 h (4.3%) was greater than the measurement error obtained from the two baseline visits in the present study (1.7%). The attenuated decline in MVC following PCM_cold_ in the present study is in line with recent studies conducted by Clifford et al. (2018) and Kwiecien et al. (2018), which displayed a substantially accelerated recovery of MVC strength in the days following soccer match-play and an eccentric based exercise protocol, respectively. The magnitude of improvement in the present study, however, was lower than that of Clifford et al. (2018), who found a treatment × time interaction and large effect size between the PCM_cold_ and PCM_amb_ condition at 36 h post-match. This could have been due to the substantially lower decline in MVC in the present study (4 ± 5% reduction at 48 h compared with ∼15% at 36 h post-match in the study by Clifford et al. (2018)), possibly owing to participants continuing to train in the days post-match and potentially compounding reductions in MVC in the study by Clifford et al. (2018), while players in the present study refrained from physical activity in the 72 h post-match. Taking this into consideration, it could be suggested that PCM could be a useful tool during periods of heavy training and/or competition, during which impairments in muscle function could be compounded by limited recovery periods.

The ability of the muscle to generate maximum force is influenced by the capacity of the CNS to activate the muscle, and the efficacy of excitation-contraction processes occurring at or distal to the neuromuscular junction to produce force in response to neural input. In the present study, motor nerve VA was greater following PCM_cold_ compared with PCM_amb_ at 48 h, while, similar to MVC, the between-treatment difference in the reduction in VA between baseline and 24 (5.0%) and 48 h (3.8%) was greater than the VA measurement error (2.8%). Considering the higher VA following PCM_cold_ coupled with the lack of effect on contractile function (Q_tw,pot_), this suggests that the beneficial effect of PCM_cold_ on MVC was primarily due to improvements in VA. It is difficult to deduce the precise mechanisms by which PCM_cold_ improved VA in the present study. However, while the mechanisms underpinning the prolonged reduction in VA post-exercise are largely unknown (Carroll et al., 2017), it is well established that muscle damage elicits impairments in VA which can take several days to resolve (Goodall et al., 2017). Factors associated with inflammation are thought to interfere with CNS function, through an increase in firing rate of group III and IV muscles afferents sensitive to various markers of muscle injury (e.g., bradykinin, histamines, and prostaglandins; Pitman and Semmler, 2012). Alternatively, or additionally, increases in the concentration of brain cytokines following eccentric exercise are potent modulators of brain function (Dantzer, 2001), and might also influence recovery of CNS impairment (Carmichael et al., 2006). Given that one of the proposed benefits of cryotherapy is to attenuate inflammation, it is possible that wearing PCM_cold_ post-match could have reduced the inflammatory response and thereby attenuated inflammation-induced perturbations in CNS function. However, given that markers of inflammation were not measured in the present study, this suggestion remains speculative. Further research examining the effects of PCM_cold_ on inflammation concurrent with measures of VA following damaging exercise is thus warranted.

A number of previous studies have shown that muscle damage leads to prolonged impairments in contractile function, as evidenced through protracted reductions in Q_tw,pot_ (Endoh et al., 2005; Goodall et al., 2017). It is likely that the prolonged reductions in Q_tw,pot_ following eccentric based exercise are a consequence of direct myofibrillar damage, disorganization of sarcomeres and interference with cellular Ca^2+^ handling which inhibit the excitation-contraction coupling process (Skurvydas et al., 2016). The lack of change in M_max_, a measure of neuromuscular transmission, suggests that the prolonged reduction in Q_tw,pot_ in the present study was due to processes beyond the sarcolemma. In addition to measuring M_max_ and Q_tw,pot_, we included measures of muscle contractility (namely, MRFD, CT, MRR, and RT_0.5_) to attempt to provide further insight into contractile function following soccer match-play. Similar to previous studies (Rampinini et al., 2011; Brownstein et al., 2017), reductions in Q_tw,pot_ persisted despite a lack of change in measures of contractility, suggesting that impairments within the excitation-contraction coupling process which were not detectable through our measures of muscle contractility were responsible for the decline in Q_tw,pot_. While we cannot discern the precise mechanism for the protracted impairment in contractile function, events that occur secondary to the initiation of muscle damage have also been implicated in impairments in excitation-contraction coupling. Specifically, the accumulation of reactive oxygen/nitrogen species has been shown to interfere with SR Ca^2+^ release, which has been attributed to redox modification of ryanodine receptors (Cheng et al., 2016), while reactive oxygen species are also thought to diminish the calcium sensitivity of myofilaments (Moopanar and Allen, 2005; Reid, 2008). In this regard, it was thought that the application of cryotherapy, which has been suggested to inhibit the inflammatory response and limit the generation of reactive oxygen/nitrogen species (White and Wells, 2013), could ameliorate the impairments in contractile function in the days following soccer match-play. However, the application of cold PCM had no effect on recovery of either Q_tw,pot_. The lack of effect of PCM_cold_ on neuromuscular function could have been due to a number of factors. Firstly, whether or not cryotherapy actually reduces inflammation remains equivocal, despite its widespread application (Broatch et al., 2014; Peake et al., 2017). Veritably, studies have neither been consistent nor produced compelling evidence to support the role of cryotherapy in reducing inflammation and improving aspects of recovery (Leeder et al., 2012), and it has been suggested that many of the previously reported benefits of cryotherapy could simply be due to a placebo effect, rather than any physiological effect (Broatch et al., 2014). Despite the promising findings from recent studies using cold PCM as a recovery aid (Clifford et al., 2018; McHugh et al., 2018), and that applying these garments has been shown to reduce muscle temperature (Kwiecien et al., 2018), there is no evidence to suggest that cold PCM reduces inflammation. As such, it is possible that PCM_cold_ had no effect on the inflammatory processes suggested to interfere with contractile and CNS function. Secondly, as alluded to previously, the magnitude of the impairments in Q_tw,pot_ was relatively small, potentially limiting the ability to detect subtle differences between groups. Indeed, it would be reasonable to assume that the benefits of cryotherapy on recovery would only be evident were the impairments in neuromuscular function more substantial than those seen in the present study. Further research to examine the effects of wearing cold PCM on recovery of neuromuscular function following exercise which elicits substantially more damage is probably warranted.

### Effect of PCM_cold_ on Physical Function

Despite the improvement in MVC and motor nerve VA with PCM_cold_, there was no improvement in measures of jump performance (CMJ or DJ-RSI). The lack of effect of PCM_cold_ on physical performance measures might have been due to the relatively modest improvement in MVC and VA in the present study. Thus, the functional relevance and meaningfulness of the differences between PCM_cold_ and PCM_amb_ for MVC and VA are unclear, and could be questioned. Although it is plausible that a reduced capacity of the CNS to activate muscles would impede the ability to perform tasks requiring maximal force production, given that current methods of determining VA are restricted to isometric or isokinetic contractions, it is not possible to accurately qualify the functional consequences of reduced VA on the performance of unconstrained physical tasks relevant to football performance.

### Limitations

This study used a competitive soccer match in order to study the effects of the application of cold PCM on recovery in the days post-match. While this approach provides the most ecologically valid means of investigating the effects of a recovery intervention following soccer match-play, one limitation of this method compared with a laboratory simulation is the lack of experimental control over the activity profiles of the players and the high inter-subject variability in match demands. Consequently, it is possible that differences between match-demands could have influenced the magnitude of fatigue and time-course of recovery following the two treatments. However, differences between the time-motion and heart rate variables between the matches were negligible. Furthermore, although simulated match protocols are designed to replicate the physiological demands of competitive matches, many of the neuromuscular, skill and cognitive demands associated with competitive match-play cannot be replicated through match simulations, and the validity of using these protocols when assessing the efficacy of a recovery intervention could thus be questioned. In addition, although no differences were found in the results from the belief questionnaires, on average, participants reported that they believed both PCM_cold_ and PCM_amb_ were “moderately effective” in improving recovery both before and following the intervention. As such, it is possible that a placebo effect could have influenced recovery under both conditions. However, the magnitude of fatigue and the time-course of recovery was similar to that observed following competitive match-play in professional soccer players (Rampinini et al., 2011), suggesting that any placebo effect on the results was negligible. Furthermore, that the participants believed both interventions to be moderately effective could be considered an important finding given that a growing body of evidence indicates that recovery is related to individual preference and perceptions of the intervention (Halson, 2014). Moreover, because local tissue temperature was not measured in the present study, it is unknown whether or not PCM_cold_ had the desired effect in regards to cooling the muscle. Nevertheless, previous work has displayed that PCM_cold_ reduced skin temperature to 22°C for 3 h following eccentric based exercise (Kwiecien et al., 2018). Thus, it is likely that the skin temperature was, similarly, decreased in the present study. Finally, another limitation of the present study was the 4–8 week gap between matches for each condition. Consequently, it is possible that players were in a different phase of the training cycle between the two matches, potentially influencing the magnitude of fatigue and time-course of recovery in response to competitive match-play. However, the majority of fixtures were separated by 6 weeks or less, with only two matches separated by 8 weeks. As such, it is likely that the influence of the duration between conditions had a negligible effect on the results of the study.

## Conclusion

The present study showed that applying cooled phase change material to the quadriceps and hamstring muscles for 3 h following soccer match-play resulted in a modest effect on the recovery of MVC and VA, but had no effect on contractile or physical function, or perceptual responses. It is possible that the lack of effect of these garments could be due to the relatively small impairments in contractile and physical function in the days post-match. Despite the limited benefit of PCM on recovery, the results from the belief questionnaires indicated that participants believed the PCM to be moderately effective in improving their recovery following the intervention. This could be considered an important finding given that the efficacy of recovery interventions could be related to individual preference and perceptions of the intervention. Further investigations are warranted to assess whether cold PCM has any effect of neuromuscular function during periods of fixture congestion, when muscle damage could be compounded by the limited recovery periods.

## Ethics Statement

This study was carried out in accordance with the recommendations of the Northumbria University Health and Life Sciences Ethics Committee, with written informed consent from all subjects. All subjects gave written informed consent in accordance with the Declaration of Helsinki. The protocol was approved by the Northumbria University Health and Life Sciences Ethics Committee.

## Author Contributions

CB, KT, SG, GH, and MM contributed to the conception and design of the work, contributed to the interpretation, and analysis of the data. CB, PA, and JŠ acquired the data for the study. All authors have drafted and revised the intellectual content and revised the final version. All listed authors qualify for authorship.

## Conflict of Interest Statement

The authors declare that the research was conducted in the absence of any commercial or financial relationships that could be construed as a potential conflict of interest.

## Footnotes

1 www.randomizer.org
